# Tobacco use: prevalence, pattern, and predictors, among those aged 15-49 years in Nigeria, a secondary data analysis

**DOI:** 10.18332/tid/82926

**Published:** 2018-03-16

**Authors:** Elias C. Aniwada, Nwachinemere D. Uleanya, Edmund N. Ossai, Emmanuel A. Nwobi, Michael Anibueze

**Affiliations:** 1 Department of Community Medicine, University of Nigeria Enugu Campus, Enugu, Nigeria; 2 Department of Paediatrics, Enugu State University Teaching Hospital, Enugu, Nigeria; 3 Department of Community Medicine, Ebonyi State University of Science and Technology, Abakaliki, Nigeria; 4 Department of Public Health Federal Ministry of Health, Abuja, Nigeria

**Keywords:** prevalence, pattern, predictors, tobacco use, Nigeria

## Abstract

**INTRODUCTION:**

Tobacco use is a major global public health challenge. It is a risk factor for most leading causes of death, and its health impacts span from conception to adulthood. This study aims to analyse tobacco use data from the 2013 Nigerian Demographic and Health Survey (NDHS), assessing the prevalence, pattern, and socio-demographic correlates of tobacco use among Nigerians aged 15-49 years.

**METHODS:**

A secondary data analysis involving 2013 NDHS was done. Data on 17 322 respondents were extracted from 36 800 participants. This number represents respondents with complete data on outcome variables of interest. Primary Sampling Unit defined on the basis of Enumeration Areas from the 2006 census was used. Head of selected household, all men and women aged 15-49 were studied. Data was collected using questionnaires. A chi-squared test and a binary logistic regression model were used in the analysis.

**RESULTS:**

Generally, 6.6% of the respondents smoked cigarettes, 1.7% used snuff, 0.4% smoked pipe, and 0.2% chewed tobacco. Based on gender, 6.6% males and 6.3% females smoked cigarettes, 0.3% males and 0.4% females smoked cigarettes as well as used snuff. Predictors of cigarette use included being in age group 25-34 years (AOR 5.8; 95% CI 4.6-7.2), being ≥35 years (AOR 4.1; 95% CI 4.1-6.8), having attained primary education (AOR 1.4; 95% CI 1.2-1.8), living in north region (AOR 1.3; 95% CI 1.1-1.5), as well as being a Moslem (AOR 0.6; 95% CI 0.5-0.7).

**CONCLUSIONS:**

A minor proportion of both genders uses tobacco with the commonest form being cigarettes. The commonest combination was cigarettes and snuff, even on stratification by gender. The identified predictors were age in categories, educational level and religion.

## INTRODUCTION

Tobacco use has reached high epidemic proportions, and is a major global public health challenge. It is a major risk factor for most leading causes of death worldwide^1^, and kills many of its users^2^. This has made tobacco the leading threat to the global economy causing loss of productivity, plus pain, grief and misery^3^. Globally, tobacco use is a major cause of avoidable and premature mortality and morbidity, accounting for about 6 million deaths^4,5^. One person dies every six seconds due to tobacco related disease. Of these deaths, 75% occur in low and middle income countries where more than 80% of the world’s smokers, including Nigeria’s, live^4^.

The death toll from tobacco is estimated to reach over 8 million deaths per year by 2030, and if unchecked, tobacco could kill over 1 billion people in the 21st century^6,7^. It is estimated that 100 million premature deaths occurred globally in the 20th century, due to tobacco smoking. Equally, tobacco smokers are believed to lose one decade of their life expectancy, when compared to people who have never smoked^8^.

The Global Adult Tobacco Survey report for Nigeria shows that 4.5 million (5.6%) adults aged 15 years or older currently smoke (10.0% men and 1.1% women), while 6.4 million (29.3%) of adults were exposed to second-hand smoke during visits to public places^11^. The Global Youth Tobacco Survey of Nigeria 2008 showed that one in five students aged 13-15 years had ever experimented with cigarette smoking, and about one in ten students currently smoked cigarettes^12^. In a study among Pharmacy students in Lagos, Nigeria, the current smoking prevalence was low at 5.5%^13^. In Nigeria, 7 in 10 current smokers planned to or were thinking about quitting; and 6 in 10 male smokers who visited a health care provider in the previous 12 months were advised to quit^11^.

Several studies have documented that tobacco use and smoking are associated with some socio-demographic factors including; age, gender, marital status, education, employment, occupation, religion, ethnicity and place of residence (urban/rural)^1-3^. A study in Nepal documented that the use of any form of tobacco was significantly associated with respondents’ age, marital status, educational status, occupation, environment and watching television. The study found that; those aged 36-49 years were about 2 times more likely to use any form of tobacco than the younger age group 15-24 years; men married or in a relationship were about 2 times more likely to use tobacco. Men with no education (laborers) were about 4 times more likely than those that had education. However, watching television at least once weekly was found to reduce the risk of smoking^3^. Another study found that prevalence amongst men was significantly higher than in women for any type of tobacco use (56.5% and 19.6%, respectivley), older adult, men, lesser education, and lower wealth quintile were more likely to use all forms of tobacco^2^.

Similarly studies also reported that poor people are more likely to smoke more, less willing to quit smoking and more likely to die from smoking than people in the highest socio-economic groups^4-7^. The same trend is observed in initiation, as the likelihood that a young person will start smoking is higher in less privileged groups^8^. The identified rising social inequality of smoking and the associated health impacts were attributable to the age of initiation of smoking^6,7,9^.

In previous studies involving the use of tobacco in Nigeria, representative data for the whole country were not used. It was either that residents in big cities or suburbs, adolescents or young adults, students in higher education institutions, or other specific population studies on cigarette smoking in limited areas, were studied. Moreover, the way the groups were sampled may have led to an overgeneralization of the findings. Equally, the economical, geographical and socio-cultural differences of these groups studied make inferences and generalizations to the entire Nigerian population difficult. Patterns of tobacco use, especially its use in different forms by the same individuals, are not usually considered. However, the Nigerian Demographic and Health Survey (NDHS) is a nationwide survey of Nigerians aged 15-59 years, and thus provides truly reliable and nationally representative data. Its analysis is expected to provide a comparable and reliable prevalence estimate for tobacco use in Nigeria. This study aimed to analyse tobacco-use data from the 2013 Nigerian Demographic and Health Survey, quantify prevalence, and describe the socio-demographic correlates of tobacco use among Nigerians aged 15-49 years.

## METHODS

### Study area

Nigeria is in sub-Saharan Africa. It is grouped into six geo-political zones including North-West, North-East, North-Central, South-West, South-East and South-Central zones. Administratively, Nigeria is divided into 36 States and the Federal Capital Territory of Abuja. Each State is made up of a number of Local Government Areas (LGAs). There are 774 LGAs in Nigeria, each is subdivided into Localities. There are widely varied regional health indices with the southern region being better than the northern region. Nigeria’s urbanization growth rate is estimated at 5.3% per year^14^. Nigeria comprises many tribes and languages.

### Study design

This is a secondary-data analysis involving the 2013 Nigeria Demographic and Health Survey. The NDHS is a cross sectional survey executed by the National Population Commission (NPC) with the main objective to provide updated estimates of basic social, demographic, economic and health indicators covering: human reproductive health, maternal and child health, awareness and behaviour regarding HIV/AIDS, other sexually transmitted infections, violence against women, and information on Tobacco use^14^.

### Sampling technique and sample size

The Primary Sampling Unit (PSU) used in the survey was defined on the basis of Enumeration Areas (EAs) from the 2006 census. During the 2006 national population census, Local Government Areas were divided into Localities, and each Locality was further subdivided into census EAs, and then clusters for convenience. Household enumeration and mapping in the selected clusters were done to produce a list of households that made up the sampling frame. The final sample size was 36 800 households selected with a minimum target of 950 completed interviews per State. A stratified, two-stage cluster design that uses Probability Proportional to Size (PPS) technique was used to identify clusters within the EAs and to choose households randomly within the clusters, achieving a nationally representative sample that appropriately includes both rural and urban residents, as well as both upper, middle and high Socio-Economic Status (SES) groups^14^. In the first stage, a total of 888 clusters (PSU), 286 in urban and 602 in rural areas, were selected by systematic sampling using the PPS technique. In the second stage, an average of 41 households were selected by equal probability systematic sampling in each cluster from a list of all private households.

### Study population/participants

The study population/participants include: head of selected household who answers questions on the household and provides a listing of household residents, as well as visitors who slept over the night before the survey; all women aged 15-49 years and men 15-59 years, who were either permanent residents of the households or visitors who stayed overnight on the night before the survey.

### Study instruments

Data collected for the 2013 NDHS involving use of questionnaires (household, women’s, and men’s questionnaires) were used. It was pretested and a standard protocol observed in administering them. These questionnaires were adapted to collect information on relevant demographic, social, economic factors and health status/indicators, as well as information about tobacco use from eligible members of the selected households. It was translated from English into three major Nigerian languages; Hausa, Igbo and Yoruba. The questionnaires were interviewer administered face-to-face with all eligible participants.

### Data analysis

Data on 17 322 respondents were extracted from 36 800 participants in the 2013 NDHS data. This number represents respondents with complete data on outcome variables of interest. Data were summarized using frequencies and percentages. Chi-squared test of statistical significance was used to verify associations of socio-demographic factors with tobacco use, while binary logistic regression model was used to identify predictors of tobacco use. The variables for regression were adjusted for one another. The level of statistical significance was determined by p<0.05.

## RESULTS

Table 1 shows the socio-demographic characteristics of respondents. The mean age of the respondents was 31.7±11.7 years. The majority of the household heads were males (90.4%). The highest proportion of respondents (48.3%) had attained secondary education. Also, the highest proportion of respondents studied were from the North-West geo-political zone (23.8%) and the least from the South-East (9.7%). There was approximately an equal distribution of religion; Christianity 51.7% and Islam 46.9%. About 50% were never in union. A higher proportion of respondents (58.8%) lived in the rural area, and approximately one-fifth of the respondents (20.4%) belonged to the middle-wealth index.

**Table 1:** Socio-demographic characteristics of respondents using 2013 NDHS

*Socio-demographic characteristics*	*Total (N=17 322) Frequency (n)*	*Per cent (%)*
**Age in categories (years)**		
15-24	6532	37.7
25-34	5119	29.6
≥35	5671	32.7
** Mean ± SD**	**31.69 ± 11.69**	
**Gender of head of household**		
Male	15662	90.4
Female	1660	9.6
**Educational level**		
No formal education	3347	19.3
Primary	2972	17.2
Secondary	8372	48.3
Higher	2631	15.2
**Region**		
North-Central	3017	17.4
North-East	2835	16.4
North-West	4121	23.8
South-East	1674	9.7
South-Central	3029	17.5
South-West	2646	15.3
**Religion**		
Christianity	8954	51.7
Islam	8120	46.9
Others (Traditionalist, Atheist)	248	1.4
**Marital status**		
Never in union	8510	49.1
Married with spouse	318	1.8
Others	8494	49.0
**Residence**		
Urban	7129	41.2
Rural	10193	58.8
**Wealth index**		
Poorer/Poorest	5663	32.7
Middle	3534	20.4
Richer/Richest	8125	46.9

Table 2 shows the prevalence and pattern of use of tobacco in different forms. About 6.6% smoked cigarettes, 1.7% used snuff, 0.4% smoked pipe, and 0.2% chewed tobacco. Of those that smoked cigarettes, 3.1% smoked pipe, 2.4% chewed tobacco, and 4.6% used snuff, in addition. The range of sticks of cigarettes they smoked was 3-10 sticks, with an average of 5.

**Table 2:** Prevalence and pattern of use of tobacco in different forms using 2013 NDHS

*Tobacco use (form)*	*Total (N=17 322) Yes Freq (%)*	*No Freq (%)*
Smoke cigarettes	1140(6.6)	16182(93.4)
Smoke pipe	61(0.4)	17261(99.6)
Chew tobacco	33(0.2)	17289(99.8)
Uses snuff	292(1.7)	17030(98.3)
Smoke cigarettes and pipe	35(0.2)	17287(99.8)
Smoke cigarettes and chew tobacco	27(0.2)	17295(99.8)
Smoke cigarettes and use snuff	53(0.3)	17269(99.7)
Smoke pipe and chew tobacco	0(0.0)	17322(100.0)
Smoke pipe and use snuff	0(0.0)	17322(100.0)
Chew tobacco and use snuff	1(0.0)	17321(100.0)
	n = 1140*	
Smoke cigarettes and pipe	35(3.1)	1105(96.9)
Smoke cigarettes and chew tobacco	27(2.4)	1113(97.6)
Smoke cigarettes and use snuff	53(4.6)	1087(95.4)
Average number of cigarette sticks in 24 hours		
*Median (IR)*^#^	*5(3-10)*	

* number that smokes cigarettes

# Interquartile range

Table 3 shows the prevalence and pattern of use of tobacco in different forms stratified by gender. About 6.6% males and 6.3% females smoked cigarettes, 1.8% males and 0.7% females used snuff, 0.4% males and 0.2% females smoked pipe, and 0.2% males and 0.4% females chewed tobacco. Of those studied, 0.2% of males and of females, smoked cigarettes and pipe, 0.1% males and 0.3% females smoked cigarettes and chewed tobacco, 0.3% males and 0.4% females smoked cigarettes and used snuff, only one male respondent chewed tobacco and used snuff, and none of the respondents smoked pipe and chewed tobacco, or smoked pipe and used snuff.

**Table 3:** Prevalence and pattern of use of tobacco in different forms stratified by gender.

	*Male*	*Female*	
**Tobacco use (form) stratified by gender**	**Yes Freq (%)**	**Yes Freq (%)**	**p value**
Smoke cigarettes	1036(6.6)	104(6.3)	0.585
Smoke pipe	58(0.4)	3(0.2)	0.215
Chew tobacco	27(0.2)	6(0.4)	0.093
Uses snuff	281(1.8)	11(0.7)	0.001
Smoke cigarettes and pipe	32(0.2)	3(0.2)	0.839
Smoke cigarettes and chew tobacco	22(0.1)	5(0.3)	0.114
Smoke cigarettes and use snuff	47(0.3)	6(0.4)	0.667
Smoke pipe and chew tobacco	NA	NA	
Smoke pipe and use snuff	NA	NA	
Chew tobacco and use snuff	1(0.0)	0(0.0)	0.745

Table 4 shows the relationship between socio-demographic characteristics and smoking of cigarettes. The respondents who were aged 25-34 years and ≥35 years were about six times (AOR 5.8; 95% CI 4.6-7.2) and five times (AOR 4.1; 95% CI 4.1-6.8), respectively, more likely to smoke cigarettes than those aged 15-24 years. Those that had primary education were about 1.4 times (AOR 1.4; 95% CI 1.2-1.8) more likely, while those that had secondary education were 1.1 times (AOR 0.9; 95% CI 0.7-1.1) less likely, to smoke cigarettes than those with no formal education. Those in the south were about 1.3 times (AOR 1.3; 95% CI 1.1-1.5) more likely to smoke cigarettes than those in the north. Moslems were about 1.7 times (AOR 0.6; 95% CI 0.5-0.7) less likely to smoke cigarettes than Christians. Middle-class were about 1.1 times (AOR 1.1; 95% CI 0.9-1.3) more likely, while the wealthier were 1.1 times (AOR 0.9; 95% CI 0.7-1.1) less likely to smoke cigarettes than the poorer class.

**Table 4:** Relationship between socio-demographic characteristics and smoke cigarettes using 2013 NDHS

Total (N=17 322)				
*Socio-demographic*	*Yes Freq (%)*	*No Freq (%)*	*Bivariate analysis x^2^ (p value)*	*Multivariate analysis AOR ( 95%CI)*
**Age categories (years)**				
15-24	133(2.0)	6399(98.0)		1
25-34	488(9.5)	4631(90.5)	352.981	5.8(4.6-7.2)
≥35	519(9.2)	5152(90.8)	(<0.001)	5.3(4.1-6.8)
**Gender**				**1**
Male	1036(6.6)	14626(93.4)	0.299	
Female	104(6.3)	1556(93.7)	(0.585)	NA
**Educational level**				
No formal education	161(4.8)	3186(95.2)		1
Primary	341(11.5)	2631(88.5)	147.885	1.4(1.2-1.8)
Secondary	508(6.1)	7864(93.9)	(<0.001)	0.9(0.7-1.1)
Higher	130(6.6)	2501(95.1)		0.5(0.4-0.7)
**Region**				
North	504(5.0)	9471(95.0)	91.578	1
South	638(8.7)	6711(91.3)	(<0.001)	1.3(1.1-1.5)
**Religion**				
Christianity	767(8.6)	8187(91.4)		1
Islam	327(4.0)	7793(96.0)	201.300	0.6(0.5-0.7)
Others	46(18.5)	202(81.5)	(<0.001)	1.9(1.3-2.7)
**Marital status**				
Never in union	378(4.4)	8132(95.6)		1
Married with spouse	50(15.7)	268(84.3)	151.403	1.3(0.9-1.9)
Others	712(8.4)	7782(91.6)	(<0.001)	0.9(0.8-1.1)
**Residence**				
Urban	479(6.7)	6650(93.3)	0.374	1
Rural	661(6.5)	9532(93.5)	(0.541)	NA
**Wealth index**				
Poorer/Poorest	320(5.7)	5343(94.3)	13.636	1
Middle	265(7.5)	3269(92.5)	(0.001)	1.1(0.9-1.3)
Richer/Richest	555(6.8)	7570(93.2)		0.9(0.7-1.1)

NA: Not Applicable

Bivariate analysis using Chi-squared test

Multivariate analysis using Binary Logistic Regression

AOR: Adjusted Odds Ratio

## DISCUSSION

The main findings from this study include: tobacco use was low, males and females use tobacco similarly, tobacco is used in both smoked and smokeless form, socio-economic and demographic factors influence tobacco use. The proportion of respondents that smoked cigarettes in this survey was low compared to other countries, especially developed and some developing countries. The finding is similar to that from the Global Adult Tobacco Survey that reported 5.6% among adults aged 15 years and above. However, other studies have documented that prevalence of tobacco use is widely varied^15^. World Health Organisation reported that the prevalence of overall current tobacco use was 15.4% (males 19.2% and females 11.1% )^20^, which is in contrast to our findings on tobacco use. The disparity may be as a result of the differences in the population studied in terms of race, economic status, and life style, as well as legislation on tobacco use. This finding, though low, should be made even lower, especially when the negative health effects of tobacco use are considered. The implication is that even though tobacco-control programs are being propagated, more effort is required in monitoring of tobacco use so as to improve the implementation of tobacco-control measures. The approach should be multi-sectoral, including the health sector, social media, and others. This has been suggested by GATS in their assertion that Services for cessation of tobacco use could be integrated into the health system, given that a large portion of smokers are ready to quit smoking^11^.

Those aged 25-34 years and ≥35 years were more likely to smoke cigarettes than those aged 15-24 years from this study. This finding of association between age and cigarette smoking is in line with many studies. A study in Zaria, Nigeria, documented age to be associated with tobacco use^16^. The finding was also supported by a study in Nepal that showed that those in age group 36-49 years were 2.4 times more likely to use any form of tobacco than the younger age group 15-24 years^17^. Similar findings have been reported in other studies^18^. However, the finding of low prevalence of smoking among those aged 15-24 years is valid, as it has been documented that the age of initiation of smoking is a key factor in cessation of smoking and in relation to the health implications of tobacco use^19^.

This study reported that those that had secondary education and above were less likely to smoke cigarettes than those with primary education and below. This is expected as education comes with enlightenment that allows the better educated to appreciate more the implications of tobacco use. Similar studies support the finding that men with no education were 3.5 more likely to smoke tobacco^20^. Similarly, while, in Ibadan, Nigeria, prevalence of smoking was higher for the higher classes^15^, in Zaria, Nigeria, the student’s social class was not associated with adolescent tobacco use^16^. A study in Brazil equally showed that the number of tobacco users with no education or less than a year schooling were twice those with 2 or more years of education^21^.

This study showed an association between socio-economic status and tobacco use but could not identify it as a predictor of tobacco use. Some studies have also found an association between socio-economic status and adolescent tobacco use, with mixed results. A survey in Brazil showed that prevalence of smoking was higher among the poor even after controlling for age, marital status, education, employment and residence^21^. While a study reported higher use among low socio-economic status^22^, another reported higher use among high socio-economic status^23^. Smoking among youth was found more in socially disadvantaged groups^24,25^. Poor people are more likely to start smoking at a younger age, smoke more, have a lower quit rate and more likely to die from smoking^26,27^. Social determinants of smoking vary between and within countries, hence addressing this equity aspect of tobacco is an important political and public health concern^20^.

Those in the southern geo-political region were more likely to smoke cigarettes than those from the north. Moreover, Moslems were less likely to smoke than Christians. This may be due to differences in religious freedom. While the northern part is predominantly Moslem, the southern part is mainly Christian. Equally, the socio-cultural differences of these population groups studied can partly be explained by the fact that in the southern part people are more educated, with women having a more liberal social life.

Marital status showed no association with tobacco use in this study. However, other studies showed significant associations between marital status and tobacco use^17,20^. In this study, smoking was slightly higher among men than in women, though not significant. This finding is in line with many other studies that reported that tobacco use was higher among males than females in both young and old^15,17,28^. Smoking prevalence among female students at the University of Lagos was zero^29^. This may be explained partly by differences in lifestyle and the occupation of men and women. The design and method used in these studies may be responsible for the differences observed, unlike other studies where a region of the country is studied. This is supported by a study in Nepal that documented association with manual work, which males are more likely to be involved in than females^13^. However, a study documented a higher prevalence of tobacco use among females^30,31^. Moreover, other studies found no significant differences between male and female tobacco use^32,33^.

Place of residence, classified as either urban or rural in this study, had no relationship with tobacco use. In contrast, several studies documented that tobacco use and cigarette smoking is associated with place of residence^17,20^. While a study reported higher use among rural residents^34^, others have reported higher use among urban residents^35^. The findings may have been different from this current study owing to differences in the economical, geographical, and socio-cultural practices of these groups.

### Limitations

It would have been better to use primary data collected by researchers, as it would have been more appropriate and representative. Equally, there could have been changes in data or findings due to the difference in time when the data were collected and the present analysis.

## CONCLUSIONS

The use of tobacco by both males and females was low. The commonest form of tobacco use was cigarettes and the least used was chewing tobacco. The commonest combination was cigarettes and snuff, even on stratification by gender. None of them smoked pipe and chewed tobacco or smoked pipe and used snuff. The identified predictors were age in categories, educational level, region and religion. These findings show that there is serious and urgent need for improved tobacco prevention and control interventions in Nigeria. Despite the low prevalence, which is good, political will and strong policies need to be put in place to stop people from starting tobacco use while encouraging current users to quit.
